# Large Language Models as Tool for Pathology Report Data Extraction: Comment

**DOI:** 10.5146/tjpath.2024.13439

**Published:** 2025-05-31

**Authors:** Hinpetch Daungsupawong, Viroj Wiwanitkit

**Affiliations:** Private Academic Consultant, Phonhong, Lao People’s Democratic Republic; Department of Research Analytics, Saveetha Dental College and Hospitals, Saveetha Institute of Medical and Technical Sciences, Saveetha University, India

Dear Editor, we would like to discuss “Large Language Models as a Rapid and Objective Tool for Pathology Report Data Extraction (1)”. According to Bolat et al., it is possible that the large massive language models might be a new useful thing in pathology. Although employing new language models to extract data from pathology reports is a promising strategy, there are a few flaws in the process that need to be fixed. The possibility of errors during the data extraction procedure is one of the main worries. Errors in the retrieved data may result from large language models, such as ChatGPT and Google Bard, misinterpreting subtle medical terms or nuances in pathology reports. Inaccurate data may seriously affect the results of research and jeopardize the validity of study findings. Additionally, as these algorithms are processing sensitive patient data, using AI for data extraction in pathology reports may give rise to privacy and security problems. Potential avenues for future research in this area might include enhancing the precision and dependability of AI-assisted data extraction in pathology reports. Addressing the shortcomings of general-purpose models such as ChatGPT and Google Bard may be possible through the development of specialized language models trained only for the medical sector. Ensuring the quality of the data can also be achieved by manually reviewing the retrieved data by pathologists to validate its accuracy. Additionally, investigating the application of natural language processing methods to decipher and comprehend pathology reports’ intricate terminology could improve the efficiency of AI algorithms for data extraction. The potential impact of AI-assisted data extraction on clinical practice is one area that is frequently disregarded in the literature. Although academic research is usually the main focus, clinical decision-making may be significantly impacted by the use of AI for pathology report processing. Artificial Intelligence (AI) has the potential to enhance patient outcomes by facilitating healthcare providers’ decision-making process by automating the extraction and analysis of data from pathology reports. The utilization of pathology reports in patient care might be completely transformed by incorporating AI technology into clinical workflows, which would ultimately result in more individualized and efficient treatment plans.

Overall, even though using AI-assisted data extraction in pathology reports has the potential to improve research speed and precision, it’s critical to address the drawbacks and difficulties that come with this methodology. Future studies should concentrate on enhancing the precision and dependability of AI algorithms, verifying the data that has been retrieved, and investigating the therapeutic applications of AI in the interpretation of pathology reports. AI technologies have the power to revolutionize pathology and enhance patient outcomes by tackling these issues. Since ChatGPT relies solely on user input from humans, programming that deals with human behavior must be carefully chosen (2,3).

## Conflict of Interest

The authors declare that they have no conflict of interest.
